# Gender Differences in the Relationship Between Sleep Problems and Suicide Attempt in Adolescents

**DOI:** 10.3389/fpsyt.2020.00133

**Published:** 2020-02-28

**Authors:** Yuhui Wan, Huiqiong Xu, Shanshan Wang, David Boyda, Danielle Mcfeeters, Ying Sun, Shichen Zhang, Ruoling Chen, Fangbiao Tao

**Affiliations:** ^1^Department of Maternal, Child and Adolescent Health, School of Public Health, Anhui Medical University, Hefei, China; ^2^Anhui Provincial Key Laboratory of Population Health & Aristogenics, Hefei, China; ^3^Faculty of Education, Health and Wellbeing, University of Wolverhampton, Wolverhampton, United Kingdom

**Keywords:** sleep problem, suicide attempt, adolescents, gender, sleep duration

## Abstract

There are few studies examining which types of sleep problems are independently associated with suicide attempt (SA) and gender difference in adolescents. The aim of the present study was to examine whether specific sleep problems were uniquely associated with suicide attempt in adolescents and explore gender differences in the association. A school-based health survey was conducted in four provinces within China from November 2014 to January 2015. A total of 15,132 students aged 10–21 years completed standard questionnaires assessing past 12 month suicide attempt in addition to measures of sleep quality, quantity and sleep beliefs. 5.4% of participants reported a suicide attempt within the last 12 months. After adjustment for sociodemographic variables and psychological symptoms, almost all sleep problems remained significantly associated with a greater endorsement of suicide attempt. Further adjustment for co-occurring sleep problems revealed that weekday sleep duration (<6, 8–10, and ≥10 h), insomnia (often), and nightmares (sometimes and often) remained independently associated with suicide attempt in boys (*p* < 0.05). However in girls, weekday sleep duration (<6 and ≥10 h), weekend sleep duration (<6 h), midday nap (0 or 1–2 d/week), insomnia (sometimes and often), nightmare (often) and sleep beliefs (high) were independently associated with suicide attempt (*p* < 0.05). Multiple sleep problems are associated with suicide attempt in adolescents, however the relationship varies by gender.

## Introduction

As the second leading cause of death in young people worldwide (1), adolescent suicide continues to exact a substantial economic, social and psychological burden for individuals, families, and communities globally. Whilst progress has been made in our understanding of the phenomenology and risk factors associated with adolescent suicidality, less is known about the role of sleep in suicidal behavior. This is despite sleep problems being linked to a myriad of other psychosocial and general health complications in adolescent populations (2).

Prevalence studies suggest that sleep problems are relatively common among adolescents (3). To date, several measures of sleep including sleep duration, sleep quality and sleep disturbances have been examined in relation to youth suicidality (4, 5). A study in Scottish secondary schools found that both insomnia symptoms and nightmares were associated with an increased likelihood of reporting suicidal ideation (6). Nevertheless, there remains enduring inconsistencies surrounding the relationship between other aspects of sleep (e.g., sleep duration) and youth suicidality (7). For example, using a student sample, Guo et al. concluded that there was a U-shaped association between sleep duration, suicidal ideation, and suicide attempt (SA). Students who reported a total sleep time of 5–7 h/day were less likely to ideate (adjusted odds ratio, AOR = 1.59) or attempt (AOR = 1.53). Conversely, students who reported sleeping <5 h/day demonstrated an increased risk of suicidal ideation (AOR = 2.28), and attempt (AOR = 3.20) but likewise students who reported sleeping >9 h/day (AOR = 2.67) also exhibited an increased likelihood of suicide attempt (8).

Moreover, trends within sleep and suicide research tend to focus on singular sleep problems in isolation (3). This has resulted in few studies examining which types of sleep problems are independently associated with SA after adjusting for the influence of other inter-correlated sleep parameters. One existing study which has differentiated the impact of multiple sleep problems, found suicide attempt (SA) to be more strongly related to the presence of nightmares than other sleep problems (difficulties initiating sleep, maintaining sleep, and early morning awakening) (9). Notwithstanding the commonalities between sleep duration and insomnia, they represent distinct sleep parameters, with divergent gender patterns and differential effects on outcomes such as school performance (10). It is therefore important to assess a wide range of sleep problems simultaneously in order to ascertain the true nature of the relationships.

Research examining gender differences in the relationships has also been mixed. Whilst some studies have reported that a stronger relationship between short sleep durations and suicide attempt in girls (11), others have concluded that short sleep durations are associated with a heightened likelihood suicidal ideation in males only (12). Yet further studies have failed to document any discernible gender differences in the relationship between nightmares and suicide risk (13). In light of apparent disparities and evidenced gender differences in sleep requirements (14), the impact of sleep problems (15) and the presentation of SA (16), this study sought to firstly investigate whether specific sleep problems were uniquely associated with SA, and secondly, to explore gender differences across these associations.

## Methods

### Study Sample and Procedures

The sample was derived from an anonymous health survey conducted from November 2014 to January 2015 involving adolescents from eight-middle schools located in 4 provinces within China. China is a vast territory with diverse geographic and economic development. In order to derive a sample broadly reflective of this diversity, eight schools (four rural and four urban) from four cities: southern Yangjiang (Guangdong province), central Xinxiang (Henan province), northern Shenyang (Liaoning province), and the western Chongqing area were selected. Collectively, these cities represent the economically developed region and the developing interior regions of the country.

In each region, eight general junior and senior schools (four from rural areas) were randomly chosen to recruit participants. As eight schools were combined junior and senior schools, only 24 schools in total were selected for inclusion into the survey. A sample of 15,713 adolescents from grades 7–12, aged 10–21 years were recruited. Of the 15,713 school adolescents, 581 (3.7%) were excluded from the study because of (1) absence from school on the day of the survey or unwillingness to respond to the questionnaire, (2) missing data through fictitious or inconsistent responses. A final sample of 15,132 (96.3%) was retained for analysis. The design and data collection procedures were approved by the Ethics Committee of Anhui Medical University (2012534). Written, informed consent was obtained from the parents of all participants in the study. Assent was obtained from all participants.

### Measurements

#### Sociodemographic Profile and Psychological Symptoms

The model was adjusted for a range of demographic and psychosocial factors known to be associated with suicide including: age, gender, urban/rural residency, parents' education level (less than junior middle school, junior middle school, senior middle school, college or more), self-perceived economic status of the family (poor, moderate, or good). Psychological symptoms in the past 3 months were evaluated using the psychological domain of the “Multidimensional Sub-health Questionnaire of Adolescents” (17), which consists of 39 questions in 3 dimensions, as follows: emotional symptoms, e.g., “Do you always feel distressed?,” conduct symptoms, e.g., “Do you always have the impulse to damage something?,” and social adaptation symptoms, e.g., “Do you always hate learning at school?.” The internal consistency of the psychological domain was satisfactory (Cronbach's α = 0.958 in present study). Psychological symptoms were dichotomized by national norm of Chinese adolescents for analysis (17).

#### Sleep Problems

##### Sleep duration

Participants were asked about their *weekday* and *weekend* sleep duration via “During the past month, on an average school day, how many hours of actual sleep do you get at night?,” “Dring the past month, on an average weekend, how many hours of actual sleep do you get at night?” In line with other research, we converted the sleep duration into 4 groups (<6, 6–8, 8–10, and ≥10 h) (18). In the analysis, the most common duration was selected as reference, thus 6–8 h was selected as the reference category for weekday sleep duration, 8–10 h was selected as the reference category for weekend sleep duration. As it is common for Chinese workplaces and schools to take a *midday nap*, the frequency of naps (0–7) per week was also surveyed. To facilitate interpretation, this was divided into three levels (None: 0, Sometimes: 1–2, Often: ≥3).

##### Sleep disturbances

Participants were asked about their experience of sleep disturbances (i.e., symptoms of insomnia, nightmare, non-restorative sleep and daytime fatigue) during the past month (19). Those who endorsed at least 1 of the 3 types of *insomnia symptoms* (difficulty initiating sleep, difficulty maintaining sleep, early awakening and difficulty resuming sleep) with a frequency of ≥3 events/week were classified as having insomnia symptoms “Often.” Individuals who endorsed a minimum of 1 type with a frequency of 1–2 events/week were categorized as having insomnia symptoms ‘Sometimes'. Those with no symptoms were defined as ‘None' (7). *Nightmare* occurrences was similarly denoted as “Often” (≥3/week), “Sometimes” (1–2/week), and “None.” *Non-restorative sleep* was indexed by one item “Did you feel refreshed after sleeping over the last month?” and *daytime fatigue* was also indexed by one item “Did you feel fatigue and easily nod off during the daytime over the last month?” Responses ranged from “Often,” “Sometimes,” and “Never.”

##### Sleep beliefs

Sleep beliefs was measured using the “Dysfunctional Beliefs and Attitudes about Sleep scale (DBAS-16) (20). The scale is a 16-item version, used to identify specific sleep-related beliefs and attitudes about sleep that may be irrational or may lead to over-concern about sleep. Each item ranges from 1 (strongly disagree) to 5 (strongly agree). There are four factors within the DBAS-16: perceived consequences of insomnia, worry/helplessness about insomnia, sleep expectations, and medication. A higher total score indicates more dysfunctional beliefs and attitudes about sleep. In the present study, the DBAS-16 evidenced good internal consistency (Cronbach alpha) of 0.894. To facilitate interpretation, the scores were divided into three levels (high: *P*_75_-*P*_100_, moderate: *P*_25_-*P*_75_, and low: *P*_0_-*P*_25_) for analyses.

#### Suicide Attempt

One question was used to assess respondent's recent history of suicide attempt, specifically, “Have you ever tried to kill yourself in the past 12 months?” (Yes/No) (21).

### Statistical Analysis

Descriptive analysis was used to outline the sociodemographic characteristics of the data (i.e., prevalence of sleep problems and suicide attempt for the total sample and for boys and girls separately). Gender differences were assessed using chi-squared test for categorical variables and one-way analysis of variance for continuous outcomes. Binomial logistic regression models were used to examine the associations between sleep problems and suicide attempt, followed by multivariate logistic regressions. In the multivariate regressions, adjustment was made for age, gender, regional areas, schools, urban/rurality, parents' education level, economic status of family and psychological symptoms. In the final model, independent sleep variables associated with suicide attempt were examined after adjusting for sociodemographic, psychosocial factors and all other sleep variables. All analyses were conducted with SPSS software, version 16.0 (SPSS Inc., Chicago, IL). A *p*-value of < 0.05 was considered statistically significant.

## Results

### Characteristics of Participants

In 15,132 participants, the mean age was 15.2 years (SD = 1.8), and 51.7% (*n* = 7,823) of the sample were girls. In terms of sleep duration, 9.8% of the sample slept <6 h at night during weekdays, while 3.3% slept more than 10 h. Four percentage reported sleeping <6 h at night during weekends, and 38.0% reported sleeping more than 10 h.

A total of 8,705 (57.7%) reported taking a midday nap 3 days or more a week. The reported rates of infrequent (i.e., sometimes) “insomnia,” “nightmare,” “non-restorative sleep,” and “daytime fatigue” were 16.6, 4.1, 15.3, and 30.2%, respectively. A total of 5.4% reported attempted suicide in the last 12 months. Gender disaggregated descriptive statistics and sociodemographic factors are shown in Table 1.

**Table 1:** Characteristics of participants by gender, *n* (%): 24 schools study, China.

**Variables**	**Total**	**Boy**	**Girl**	***p*-value**
Age (mean, SD)	15.2 (1.8)	15.2 (1.8)	15.2 (1.7)	0.071
Regional areas				
Yangjiang	3,648 (24.1)	1,866 (25.5)	1,782 (22.8)	0.001
Shenyang	3,252 (21.5)	1,558 (21.3)	1,694 (21.7)	
Xinxiang	4,092 (27.0)	1,909 (26.1)	2,183 (27.9)	
Chongqing	4,140 (27.4)	1,981 (27.1)	2,159 (27.6)	
Urban/Rurality				
Urban	7,405 (48.9)	3,675 (50.2)	3,730 (47.7)	0.002
Rural	7,727 (51.1)	3,936 (49.8)	4,088 (52.3)	
Father's education level				
College or more	2,096 (13.9)	1,138 (15.6)	958 (12.3)	<0.001
Senior middle school	4,380 (28.9)	2,067 (28.3)	2,313 (29.6)	
Junior middle school	6,411 (42.4)	3,030 (41.4)	3,381 (43.2)	
Less than junior middle school	2,245 (14.8)	1,079 (14.8)	1,166 (14.9)	
Mother's education level				
College or more	1,698 (11.2)	915 (12.5)	783 (10.0)	<0.001
Senior middle school	4,146 (27.4)	2,027 (27.7)	2,119 (27.1)	
Junior middle school	6,501 (43.0)	3,034 (41.5)	3,467 (44.3)	
Less than junior middle school	2,787 (18.4)	1,338 (18.3)	1,449 (18.5)	
Economic status of family				
Good	1,877 (12.4)	992 (13.6)	885 (11.3)	<0.001
Moderate	1,1125 (73.5)	5,102 (69.8)	6,023 (77.0)	
Poor	2,130 (14.1)	1,220 (16.7)	910 (11.6)	
Psychological symptoms				
Yes	4,222 (27.9)	2,071 (28.3)	2,151 (27.5)	0.272
No	10,910 (72.1)	5,243 (71.7)	5,667 (72.5)	
Weekday sleep duration (/night)				
<6 h	1,482 (9.8)	725 (9.9)	757 (9.7)	<0.001
6–8 h	8,917 (58.9)	3,955 (54.1)	4,962 (63.5)	
8–10 h	4,236 (28.0)	2,319 (31.7)	1,917 (24.5)	
≥10 h	497 (3.3)	315 (4.3)	182 (2.3)	
Weekend sleep duration(/night)				
<6 h	604 (4.0)	402 (5.5)	202 (2.6)	<0.001
6–8 h	2,117 (14.0)	1,130 (15.4)	987 (12.6)	
8–10 h	6,662 (44.0)	3,065 (41.9)	3,597 (46.0)	
≥10 h	5,749 (38.0)	2,717 (37.1)	3,032 (38.8)	
Midday nap (day/week)				
None	4,256 (28.1)	2,281 (31.2)	1,975 (25.3)	<0.001
Sometime	2,171 (14.3)	1,006 (13.8)	1,165 (14.9)	
Often	8,705 (57.7)	4,027 (55.1)	4,678 (59.8)	
Insomnia				
None	6,792 (44.9)	3,486 (47.7)	3,306 (42.3)	<0.001
Sometime	5,826 (38.5)	2,586 (35.4)	3,240 (41.4)	
Often	2,514 (16.6)	1,242 (17.0)	1,272 (16.3)	
Nightmare				
None	11,293 (74.6)	5,692 (77.8)	5,601 (71.6)	<0.001
Sometime	3,223 (21.3)	1,320 (18.0)	1,903 (24.3)	
Often	616 (4.1)	302 (4.1)	314 (4.0)	
Non-restorative sleep				
None	7,810 (51.6)	4,070 (55.6)	3,740 (47.8)	<0.001
Sometime	5,008 (33.1)	2,173 (29.7)	2,835 (36.3)	
Often	2,314 (15.3)	1,071 (14.6)	1,243 (15.9)	
Daytime fatigue				
None	1,315 (8.7)	805 (11.0)	510 (6.5)	<0.001
Sometime	9,244 (61.1)	4,405 (60.2)	4,839 (61.9)	
Often	4,573 (30.2)	2,104 (28.8)	2,469 (31.6)	
Sleep beliefs				
High	3,897 (25.8)	2,039 (27.9)	1,858 (23.8)	<0.001
Moderate	7,395 (48.9)	3,372 (46.1)	4,023 (51.5)	
Low	3,840 (25.4)	1,903 (26.0)	1,937 (24.8)	
Suicide attempt (SA)				
Yes	817 (5.4)	371 (5.1)	446 (5.7)	0.085
No	14,315 (94.6)	6,943 (94.9)	7,372 (94.3)	

### Sleep Problems and SA

As shown in Table 2, the unadjusted model showed that various sleep problems were significantly associated with an increased risk of suicide attempt. After adjustment for gender, age, regional areas, urban/rurality, parent's education level, economic status of family, psychological symptoms, almost all of the sleep variables remained significantly associated with an increased likelihood of SA, although the associations were attenuated.

**Table 2:** Number, % and OR of SA by sleep problems: 24 schools study, China.

**Sleep problems**	***n* (%)**	**Unadjusted analysis**		**Adjusted analysis^a^**		**Adjusted analysis^b^**	
		**OR (95%CI)**	***P*-value**	**OR (95%CI)**	***P*-value**	**OR (95% CI)**	***P*-value**
Weekday sleep duration							
<6 h	208 (14.0)	3.68 (3.08–4.40)	<0.001	2.82 (2.33–3.41)	<0.001	**2.17 (1.76**–**2.67)**	**<0.001**
6–8 h	379 (4.3)	1.0		1.0		1.0	
8–10 h	178 (4.2)	0.99 (0.82–1.19)	0.898	1.17 (0.96–1.42)	0.132	**1.27 (1.03–1.56)**	**0.025**
≥10 h	52 (10.5)	2.63 (1.94–3.57)	<0.001	2.98 (2.14–4.14)	<0.001	**3.00 (2.13–4.24)**	**<0.001**
Weekend sleep duration							
<6 h	85 (14.1)	3.67 (2.83–4.74)	<0.001	2.52 (1.91–3.36)	<0.001	**1.56 (1.15–2.13)**	**0.005**
6–8 h	137 (6.5)	1.55 (1.26–1.91)	<0.001	1.40 (1.13–1.74)	0.002	**1.26 (1.01–1.58)**	**0.044**
8–10 h	285 (4.3)	1.0		1.0		1.0	
≥10 h	310 (5.4)	1.28 (1.08–1.50)	0.004	1.18 (0.99–1.39)	0.066	0.99 (0.82–1.18)	0.876
Midday nap (day/week)							
None	280 (6.6)	1.42 (1.21–1.66)	<0.001	1.55 (1.29–1.87)	<0.001	**1.43 (1.18–1.72)**	**<0.001**
Sometime	125 (5.8)	1.23 (1.00–1.51)	0.049	1.34 (1.08–1.67)	0.009	**1.27 (1.01–1.59)**	**0.038**
Often	412 (4.7)	1.0		1.0		1.0	
Insomnia							
None	219 (3.2)	1.0		1.0		1.0	
Sometime	321 (5.5)	1.75 (1.47–2.09)	<0.001	1.49 (1.24–1.79)	<0.001	**1.36 (1.12–1.66)**	**0.002**
Often	277 (11.0)	3.72 (3.09–4.47)	<0.001	2.21 (1.82–2.69)	<0.001	**1.52 (1.22–1.90)**	**<0.001**
Nightmare							
None	436 (3.9)	1.0		1.0		1.0	
Sometime	242 (7.5)	2.02 (1.72–2.38)	<0.001	1.59 (1.35–1.89)	<0.001	**1.39 (1.16–1.67)**	**<0.001**
Often	139 (22.6)	7.26 (5.87–8.97)	<0.001	3.75 (2.99–4.71)	<0.001	**2.41 (1.86–3.12)**	**<0.001**
Non–restorative sleep							
None	262 (3.4)	1.0		1.0		1.0	
Sometime	280 (5.6)	1.71 (1.44–2.03)	<0.001	1.34 (1.12–1.60)	0.002	1.14 (0.94–1.39)	0.182
Often	275 (11.9)	3.89 (3.26–4.63)	<0.001	1.98 (1.64–2.40)	<0.001	1.19 (0.95–1.49)	0.140
Daytime fatigue							
None	74 (4.0)	1.0		1.0		1.0	
Sometime	358 (3.9)	0.97 (0.75–0.1.25)	0.800	0.93 (0.72–1.22)	0.610	0.92 (0.69–1.22)	0.535
Often	385 (9.6)	2.08 (1.58–2.73)	<0.001	1.52 (1.16–2.00)	0.003	1.17 (0.87–1.58)	0.308
Sleep beliefs							
High	350 (9.0)	2.69 (2.19–3.29)	<0.001	1.55 (1.25–1.93)	<0.001	**1.26 (1.00–1.58)**	**0.049**
Moderate	331 (4.5)	1.28 (1.04–1.56)	0.019	1.01 (0.82–1.25)	0.898	1.03 (0.83–1.29)	0.763
Low	136 (3.5)	1.0		1.0		1.0	

*SA, suicidal attempt*.

a *Adjusted for gender, age, regional areas, urban/rurality, parent's education level, economic status of family, psychological symptoms*.

b *Adjusted for gender, age, regional areas, urban/rurality, parent's education level, economic status of family, psychological symptoms, all sleep variables. P < 0.05 were in bold*.

After entering sociodemographic factors and sleep variables into the final model, weekday sleep duration (<6 h, OR = 2.17, 95%CI = 1.76–2.67; 8–10 h, OR = 1.27, 95%CI = 1.03–1.56; ≥10 h, OR = 3.00, 95%CI = 2.13–4.24), weekend sleep duration (<6 h, OR = 1.56, 95%CI = 1.15–2.13; 6–8 h, OR = 1.26, 95%CI = 1.01–1.58), midday nap (none, OR = 1.43, 95%CI = 1.18–1.72; sometime, OR = 1.27, 95%CI = 1.01–1.59), insomnia (sometime, OR = 1.36, 95%CI = 1.12–1.66; often, OR = 1.52, 95%CI = 1.22–1.90), nightmare (sometime, OR = 1.39, 95%CI = 1.16–1.67; often, OR = 2.41, 95%CI = 1.86–3.12) and sleep beliefs (high, OR = 1.26, 95%CI = 1.00–1.58) were independently associated with past 12 month suicide attempt.

### Sleep Problems and SA by Gender

Table 3 shows the relationships between sleep problems and SA in male adolescents. The final adjusted model revealed that weekday sleep duration (<6 h, OR = 2.75, 95%CI 2.02–3.75; 8–10 h, OR = 1.52, 95%CI = 1.12–2.06; ≥10 h, OR = 3.82, 95%CI = 2.44–5.98), insomnia (often, OR = 1.60, 95%CI = 1.15–2.22) and nightmare (sometime, OR = 1.73, 95%CI = 1.31–2.28; often, OR = 2.63, 95%CI = 1.79–3.85) were independently associated with SA.

**Table 3:** Number, % and OR of SA by sleep problems in boys: 24 schools study, China.

**Sleep problems**	***n* (%)**	**Unadjusted analysis**		**Adjusted analysis^a^**		**Adjusted analysis^b^**	
		**OR (95%CI)**	***P*-value**	**OR (95%CI)**	***P*-value**	**OR (95% CI)**	***P*-value**
Weekday sleep duration							
<6 h	107 (14.8)	4.62 (3.54–6.01)	<0.001	3.50 (2.65–4.63)	<0.001	**2.75 (2.02–3.75)**	**<0.001**
6–8 h	143 (3.6)	1.0		1.0		1.0	
8–10 h	86 (3.7)	1.03 (0.78–1.35)	0.850	1.39 (1.03–1.86)	0.031	**1.52 (1.12–2.06)**	**0.008**
≥10 h	35 (11.1)	3.33 (2.26–4.92)	<0.001	3.59 (2.36–5.46)	<0.001	**3.82 (2.44–5.98)**	**<0.001**
Weekend sleep duration							
<6 h	54 (13.4)	4.09 (2.90–5.77)	<0.001	2.52 (1.74–3.65)	<0.001	1.40 (0.92–2.12)	0.117
6–8 h	73 (6.5)	1.82 (1.35–2.47)	<0.001	1.58 (1.16–2.17)	0.004	1.38 (0.99–1.90)	0.055
8–10 h	112 (3.7)	1.0		1.0		1.0	
≥10 h	132 (4.9)	1.35 (1.04–1.74)	0.023	1.26 (0.96–1.65)	0.092	0.99 (0.75–1.32)	0.970
Midday nap (day/week)							
None	118 (5.2)	1.04 (0.82–1.31)	0.751	1.24 (0.95–1.62)	0.123	1.15 (0.87–1.51)	0.332
Sometime	52 (5.2)	1.04 (0.76–1.42)	0.818	1.18 (0.85–1.64)	0.334	1.12 (0.80–1.58)	0.511
Often	201 (5.0)	1.0		1.0		1.0	
Insomnia							
None	106 (3.0)	1.0		1.0		1.0	
Sometime	130 (5.0)	1.69 (1.30–2.19)	<0.001	1.45 (1.11–1.91)	0.007	1.35 (1.00–1.83)	0.053
Often	135 (10.9)	3.89 (2.99–5.06)	<0.001	2.33 (1.76–3.08)	<0.001	**1.60 (1.15–2.22)**	**0.005**
Nightmare							
None	196 (3.4)	1.0		1.0		1.0	
Sometime	108 (8.2)	2.50 (1.96–3.19)	<0.001	1.92 (1.49–2.47)	<0.001	**1.73 (1.31–2.28)**	**<0.001**
Often	67 (22.2)	8.00 (4.96–8.91)	<0.001	4.22 (3.03–5.88)	<0.001	**2.63 (1.79–3.85)**	**<0.001**
Non-restorative sleep							
None	135 (3.3)	1.0		1.0		1.0	
Sometime	113 (5.2)	1.60 (1.24–2.06)	<0.001	1.22 (0.94–1.60)	0.141	1.01 (0.75–1.37)	0.925
Often	123 (11.5)	3.78 (2.93–4.88)	<0.001	1.88 (1.42–2.48)	<0.001	1.04 (0.74–1.46)	0.816
Daytime fatigue							
None	43 (3.9)	1.0		1.0		1.0	
Sometime	155 (3.5)	0.91 (0.64–1.28)	0.585	0.89 (0.62–1.27)	0.527	0.97 (0.66–1.42)	0.863
Often	173 (9.5)	2.58 (1.83–3.64)	<0.001	1.48 (1.03–2.14)	0.035	1.18 (0.79–1.79)	0.419
Sleep beliefs							
High	170 (8.3)	2.19 (1.66–2.89)	<0.001	1.23 (0.91–1.66)	0.177	1.03 (0.75–1.41)	0.855
Moderate	125 (3.7)	0.93 (0.69–1.24)	0.602	0.76 (0.56–1.03)	0.074	0.80 (0.58–1.10)	0.172
Low	76 (4.0)	1.0		1.0		1.0	

*SA, suicidal attempt*.

a *Adjusted for age, regional areas, urban/rurality, parent's education level, economic status of family, psychological symptoms*.

b *Adjusted for age, regional areas, urban/rurality, parent's education level, economic status of family, psychological symptoms, all sleep variables. P < 0.05 were in bold*.

Table 4 shows the relationships between sleep problems and SA in female adolescents. Results indicated that weekday sleep duration (<6 h, OR = 1.83, 95%CI = 1.37–2.43; ≥10 h, OR = 2.30, 95%CI = 1.29–4.09), weekend sleep duration (<6 h, OR = 1.76, 95%CI = 1.09–2.85), midday nap (none, OR = 1.72, 95%CI = 1.32–2.24; sometime, OR = 1.39, 95%CI = 1.03–1.89), insomnia (sometime, OR = 1.36, 95%CI 1.05–1.77; often, OR = 1.45, 95%CI = 1.07–1.97), nightmare (often, OR = 2.23, 95%CI 1.57–3.18), and sleep beliefs (high, OR = 1.61, 95%CI = 1.15–2.25) were independently associated with SA.

**Table 4:** Number, % and of SA by sleep problems in girls: 24 schools study, China.

**Sleep problems**	***n* (%)**	**Unadjusted analysis**		**Adjusted analysis^a^**		**Adjusted analysis^b^**	
		**OR (95%CI)**	***P*-value**	**OR (95%CI)**	***P*-value**	**OR (95% CI)**	***P*-value**
Weekday sleep duration							
<6 h	101 (13.3)	3.08 (2.41–3.95)	<0.001	2.39 (1.84–3.10)	<0.001	**1.83 (1.37–2.43)**	**<0.001**
6–8 h	236 (4.8)	1.0		1.0		1.0	
8–10 h	92 (4.8)	1.01 (0.78–1.29)	0.940	1.03 (0.78–1.35)	0.842	1.11 (0.83–1.46)	0.486
≥10 h	17 (9.3)	2.06 (1.23–3.46)	0.006	2.45 (1.42–4.24)	0.003	**2.30 (1.29–4.09)**	**0.005**
Weekend sleep duration							
<6 h	31 (15.3)	3.59 (2.38–5.42)	<0.001	2.66 (1.71–4.12)	<0.001	**1.76 (1.09–2.85)**	**0.022**
6–8 h	64 (6.5)	1.37 (1.02–1.85)	0.036	1.28 (0.94–1.74)	0.118	1.17 (0.86–1.61)	0.322
8–10 h	173 (4.8)	1.0		1.0		1.0	
≥10 h	178 (5.9)	1.23 (1.00–1.53)	0.055	1.13 (0.90–1.41)	0.301	0.97 (0.77–1.23)	0.818
Midday nap (day/week)							
None	162 (8.2)	1.89 (1.53–2.34)	<0.001	1.86 (1.44–2.40)	<0.001	**1.72 (1.32–2.24)**	**<0.001**
Sometime	72 (6.3)	1.42 (1.08–1.86)	0.013	1.46 (1.08–1.96)	0.013	**1.39 (1.03–1.89)**	**0.032**
Often	211 (4.5)	1.0		1.0		1.0	
Insomnia							
None	113 (3.4)	1.0		1.0		1.0	
Sometime	191 (5.9)	1.77 (1.40–2.25)	<0.001	1.53 (1.20–1.96)	0.001	**1.36 (1.05–1.77)**	**0.022**
Often	142 (11.2)	3.55 (2.75–4.59)	<0.001	2.14 (1.63–2.82)	<0.001	**1.45 (1.07–1.97)**	**0.016**
Nightmare							
None	240 (4.3)	1.0		1.0		1.0	
Sometime	134 (7.0)	1.69 (1.36–2.10)	<0.001	1.40 (1.12–1.76)	0.003	1.20 (0.95–1.53)	0.129
Often	72 (22.9)	6.65 (4.96–8.91)	<0.001	3.43 (2.50–4.70)	<0.001	**2.23 (1.57–3.18)**	**<0.001**
Non-restorative sleep							
None	127 (3.4)	1.0		1.0		1.0	
Sometime	167 (5.9)	1.78 (1.41–2.26)	<0.001	1.48 (1.16–1.89)	0.002	1.28 (0.98–1.68)	0.071
Often	152 (12.2)	3.96 (3.10–5.07)	<0.001	2.12 (1.63–2.78)	<0.001	1.34 (0.99–1.83)	0.062
Daytime fatigue							
None	31 (4.1)	1.0		1.0		1.0	
Sometime	203 (4.1)	1.00 (0.68–1.48)	0.987	0.99 (0.67–1.48)	0.970	0.86 (0.56–1.31)	0.478
Often	212 (9.8)	2.52 (1.71–3.71)	0.001	1.61 (1.07–2.44)	0.024	1.13 (0.72–1.78)	0.582
Sleep beliefs							
High	180 (9.7)	3.36 (2.49–4.53)	<0.001	2.04 (1.48–2.81)	<0.001	**1.61 (1.15–2.25)**	**0.006**
Moderate	206 (5.1)	1.69 (1.26–2.26)	<0.001	1.36 (1.01–1.85)	0.046	1.36 (0.99–1.86)	0.053
Low	60 (3.1)	1.0		1.0		1.0	

*SA, suicidal attempt*.

a *Adjusted for age, regional areas, urban/rurality, parent's education level, economic status of family, psychological symptoms*.

b *Adjusted for age, regional areas, urban/rurality, parent's education level, economic status of family, psychological symptoms, all sleep variables. P < 0.05 were in bold*.

## Discussion

The present study used data from a large-school survey conducted in China to investigate whether specific sleep problems were uniquely associated with SA and whether the relationship varied by gender. Our analysis suggests that various sleep problems are associated with suicide attempt in adolescents with extended weekday sleep durations and frequent nightmares shown to be among the strongest risk factors. Furthermore, results showed some discernible gender differences. Whilst sleep duration, insomnia, and nightmares were associated with suicide risk across genders, weekend sleep duration, midday nap, and sleep beliefs only reached significance within female adolescents. Moreover, although there was a broadly similar pattern observed for weekday sleep duration, insomnia and nightmare; there were slight variations in the strength and the presentation of the effects. For instance, both low and high weekday sleep durations were more strongly associated with SA in male adolescents. Moreover, even infrequent occurrences of nightmares (i.e., sometimes) were found to be associated with an elevated risk of suicide attempt amongst male adolescents, while only more frequent nightmare occurrences (i.e., often) reached significance within females. In contrast, infrequent episodes of insomnia were significant risk factors for suicide attempt among females but not males.

### Sleep Duration

We observed a similar U-shaped association between weekday sleep duration and suicide attempts that has been demonstrated in previous studies within adolescent populations (7, 8, 22, 23). In general, existing studies have indicated that short sleep durations increase the risk of suicidal behaviors, although there is currently no consensus on the definition of “short sleep” across published literature (24, 25). However, our finding that extended weekday sleep durations are associated with a higher suicide risk diverge from Gangwisch et al.'s conclusion that long sleep durations had no significant relationship with suicidal behaviors (26, 27). Further exploration of the relationship between long sleep durations and SA are warranted.

The relationship between weekday sleep duration and suicide attempt was found to be marginally stronger for boys compared to girls, but no significant difference was found. This conflicts with earlier research which has shown distinguishable gender associations. For instance, Meijer et al. reported that short sleep durations were more strongly associated with suicidal behaviors in boys than in girls (23, 28). In contrast, a web-based survey in Korean adolescents found that short sleep durations had a stronger relationship with SA in girls than in boys (11). Nevertheless, the current study extends existing knowledge by demonstrating that low weekend sleep duration and fewer midday naps also increases the risk of suicide attempt, but only in girls. By implication, the relationship between gender, sleep duration and suicide attempt require further examination.

### Sleep Distsurbances

The majority of previous studies which have investigated the role of sleep disturbance in suicidal behaviors have centered on insomnia and nightmares specifically (29–31). Uniquely, we chose to utilize a more comprehensive measure of sleep disturbance encompassing non-restorative sleep and daytime fatigue in addition to traditional assessment measures. The results suggest that insomnia and nightmares are stronger risk factors of suicide attempt than some of the other types of sleep disturbance. These findings support Wong et al.'s results that after controlling for nightmares, overtiredness and other variables, those who had trouble sleeping in early adolescence were more likely than those without trouble sleeping to think about killing oneself in late adolescence (32). Our findings also corroborate prospective research which indicates that persistent nightmares are a dominant risk factor for repeated suicide attempts over and above persistent difficulties maintaining sleep and early morning awakening (33). Future studies should continue to simultaneously model multiple sleep variables on suicide attempt in order to fully comprehend the distinctive role of specific forms of sleep disturbance.

The independent effects of insomnia and nightmare on SA were confirmed across genders in the current study. However, research examining gender differences in the relationship between sleep problems and suicidality and related risk factors (e.g., depression) has been mixed in previous studies (13, 15). Marinova et al. observed no significant gender differences in a sample of depressed patients when examining the link between nightmares and suicide attempt (13). One study in Norwegian adolescents found a significant relationship between insomnia and depression for both genders, but found the results to be stronger among boys (15). Further studies may look to clarify the comparative influence of sleep disturbances on SA in boys, distinct from girls.

### Sleep Beliefs

Unlike the research interest surrounding sleep duration and sleep disturbances in adolescent suicidality, little attention has been directed toward the role of sleep beliefs in adolescent suicide attempt. Of the few existing studies which have addressed this area, a link between more dysfunctional sleep beliefs and risk of suicidality has been observed (34–36). One qualitative interview study indicated that those with dysfunctional beliefs about sleep were more likely to report dissatisfaction with their sleep as well as increased negative thinking, attention difficulties and inactivity, which in turn led to suicidal thoughts and behaviors (36). A significant relationship between sleep beliefs and suicidal ideation was found in an adolescent patients sample (34). McCall et al. also showed that dysfunctional beliefs and attitudes about sleep mediated the relationship between symptoms of insomnia and suicidal ideation (35). The current study therefore builds upon the literature by showing that dysfunctional sleep beliefs are associated with an increased risk of suicide attempt but only in girls. Due to the scarcity of research in this area, replication studies are needed before definitive conclusions can be drawn.

### Strength and Limitation

The main strengths of this study include (i) a large representative sample from both junior and senior middle schools, and from urban and rural areas across four distinct regions in China (ii) a high response rate (96.3%), (iii) a comprehensive assessment of a variety of sleep problems, including sleep duration, sleep disturbance and sleep beliefs. Furthermore, we strengthen the limited literature by examining gender differences in the links between sleep problems and adolescent suicide attempt which has thus far been a particularly neglected area of research.

That said, the study has some potential limitations worth discussing. First, the study was cross-sectional therefore it is difficult to establish a causal relationship between sleep problems and suicide attempt. It is equally probable that the observed relationship reflects the impact of poor mental health on sleeping difficulties. Nonetheless, our conclusions linking sleep problems to suicide attempt were similar to those documented in previous prospective studies (36, 37). Second, our study only included self-report questionnaire measures, which may have contributed to mono-informant biases. Although it was impractical to assess sleep via experimental methods in large-scale population-based studies, self-reported sleep behavior has been shown to have good concordance with objectively-measured sleep (38, 39). Accordingly, future studies may seek to incorporate objective measures of sleep functioning (e.g., actigraphy) and SA. Third, previous studies have shown that physical illnesses, chronic conditions and circadian chronotype are also important predictors of adolescent sleep outcomes with the exception of sleep duration and quality (40, 41). Due to the lack of available data, we were unable to adjust for these factors in the current study which may have the effect of obscuring the accurate nature of the relationship between sleep and suicidality, thus, subsequent research should look to include a measure of youth circadian chronotype and physical ailments. Furthermore, despite the large sample, the failure to include a weighting system which takes account of the multi-stage sampling design and adjusts for sample selection within geographical region and school as well as non-response and differential socio-demographic distributions, the possibility of selection must be acknowledged as a weakness, thus caution should be exercised in extrapolating the findings to the wider population of adolescents in China. The extent to which these findings can be generalized to adolescents in other countries or cultures is also unclear as all participants in this study were located in mainland China. Finally, the results of the current study represent a preliminary investigation into the relationship between sleep problems and SA. Whilst initial work has been conducted to better understand the theoretical mechanisms underpinning insomnia and suicide associations (42), this work needs to be expanded to help account for the link between suicidal behaviors and other sleep parameters.

## Implication

An increased focus on the role of sleep problems is needed at multiple levels in order to optimize mental health outcomes. Sleep and potentially even mental health problems could be enhanced through improvements in adolescents' physical and psychosocial conditions, controlling visual screen exposure, improving sleep hygiene and daytime behaviors, and altering parents' sleep habits (43, 44). In addition, alleviating the burden of homework and tailoring school timetables could reduce circadian misalignment, along with multiple positive mental health and cognitive benefits (45, 46). As sleep is a modifiable suicide risk factor, it is important to fully explore sleep patterns in screening and assessment processes (31). Healthy sleep has been recommended as an important protective factor in the prevention of suicide attempt in adolescents (47). There is evidence that cognitive behavioral therapy for insomnia could be used to target suicide risk prevention and intervention (31). Moreover, dysfunctional beliefs and high concern about sleep offer potential targets for psychotherapy of mental health and suicidal behavior (34, 48). In any event, further studies are required to determine whether sleep screening/interventions, incorporating global sleep problems or more specific sleep domains, are effective in suicide treatment or prevention.

## Data Availability Statement

The datasets generated for this study are available on request to the corresponding author.

## Ethics Statement

The design and data collection procedures were approved by the Ethics Committee of Anhui Medical University (2012534). Written, informed consent was obtained from the parents of all participants in the study.

## Author Contributions

YW obtained funding, reviewed the topic related literature, and drafted the first version of the manuscript. HX, SW, SZ, YS, and FT performed the study design, coordination and data collection. YW and RC worked on data analysis. YW, DB, DM, and RC were involved in the interpretation of the data and revision of the manuscript. FT performed the study design and carried out study supervision and revision of the manuscript. All authors checked and interpreted the results and approved the final version. RC and FT are the guarantors for the study.

### Conflict of Interest

The authors declare that the research was conducted in the absence of any commercial or financial relationships that could be construed as a potential conflict of interest.
